# Pore Engineering for High Performance Porous Materials

**DOI:** 10.1021/acscentsci.3c00916

**Published:** 2023-08-10

**Authors:** Dongyuan Zhao, Tiancong Zhao

At the micro- and nanoscale, pores represent a highly distinctive feature, and materials containing pores are referred to as porous materials. The pore structure imparts materials with a high specific surface area, enhances mass transfer efficiency, and alters the interfacial interactions between the material host and its surrounding environment, thereby bringing about significant changes in material performances. To date, porous materials represented by mesoporous materials^1^ and metal-/covalent-organic frameworks (MOF/COF)^2^ have made outstanding contributions to the development of numerous fields. Due to the significance of porous materials, the engineering and application of pores have always been a research area of great importance. This virtual issue of *ACS Central Science* highlights the latest research on porous materials published in this journal recently. We have selected 18 representative research achievements and provide a brief introduction of their work and porous materials.

**Scheme 1:**
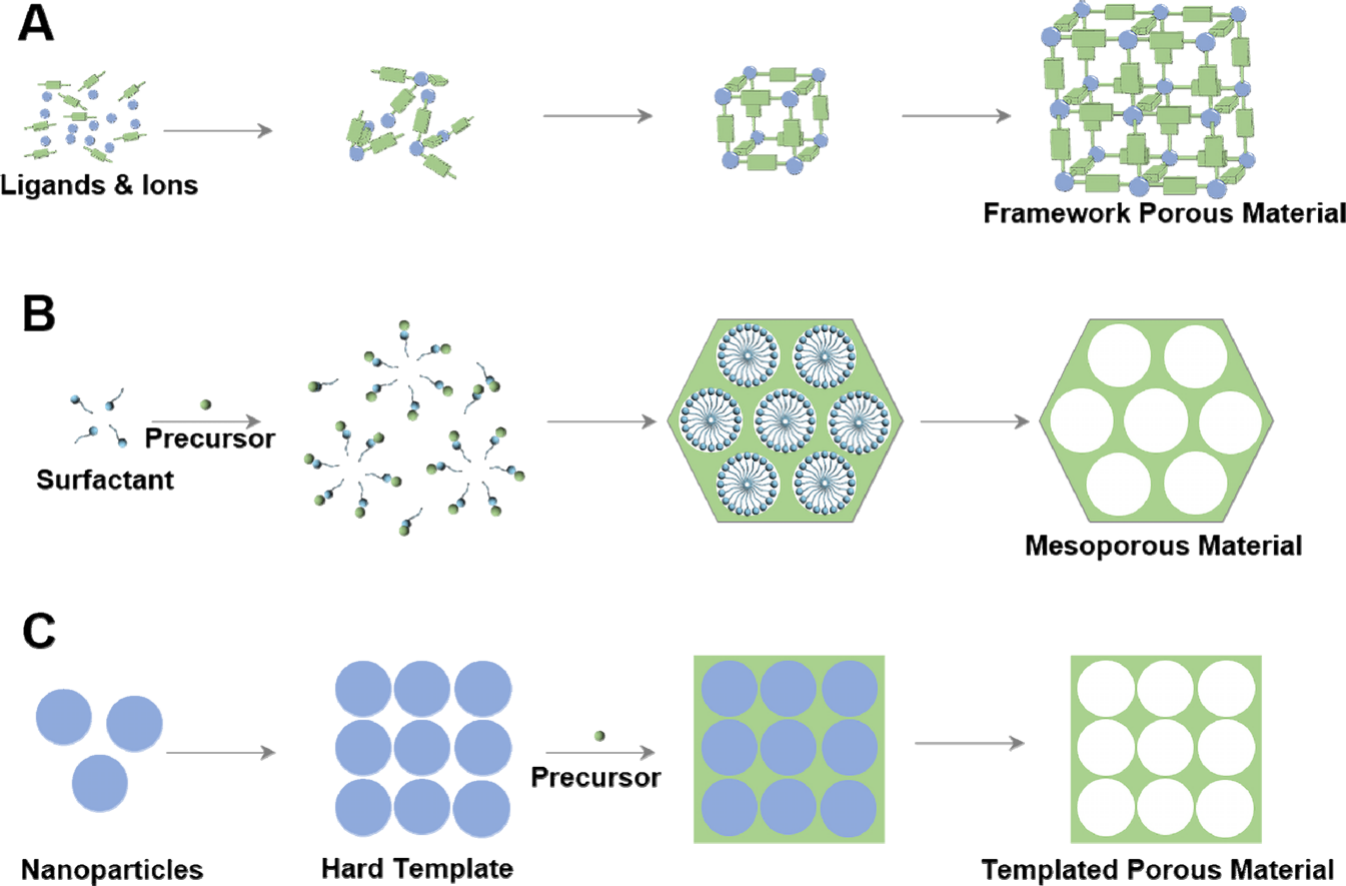
Schematic Illustration of the Fabrication Process of (A) Framework Porous Materials, (B) Mesoporous Materials, and (C) Hard-Templated Based Microporous Materials

Porous materials can be classified into microporous, mesoporous, and macroporous materials based on pore size, and their synthesis methods include self-templating, soft-templating, hard-templating, and so on. The key scientific issues in current porous materials research can be divided into two categories: (1) engineering of pore structures; (2) investigation of the relationship between pore structures and material properties. Different synthesis methods involve different ways of constructing pore structures, which can result in distinct properties. The articles selected for this virtual issue have made outstanding contributions and breakthroughs in these two areas. Brief introductions for each of the 18 articles in this virtual issue are presented below. These articles are categorized based on the challenges they address: construction of microporous frameworks, synergistic assembly of mesopore channels, templating methods for macropore formation, the influence of pore structures on mass transfer, and the influence of pore structures on catalytic performance.

## Pore Engineering of Framework Structures

Porous framework materials, represented by metal–organic frameworks (MOFs) and covalent organic frameworks (COFs), rely on the assembly of organic ligands with metal ions or organic ligands themselves to form ordered framework structures. They have broad application prospects in areas such as adsorption, catalysis, and energy.^3^ However, due to the highly ordered framework structures and strong coordination between ligands, it is challenging to engineer the pores of framework materials.

Omar M. Yaghi and his colleagues employed a linker extension strategy to construct a novel MOF with enhanced water harvesting ability.^4^ They selected MOF-303, which uses 1*H*-pyrazole-3,5-dicarboxylate (PZDC) as the ligand unit, and introduced a molecule called (*E*)-5-(2-carboxylatovinyl)-1*H*-pyrazole-3-carboxylate, which has an additional ethylene group, to replace PZDC. The introduction of the ethylene group simply extended the length of the ligand, leading to a larger pore volume without changing the hydrophilic–hydrophobic pocket environment of the pores. As a result, they successfully achieved an increased pore volume in MOF-303 without compromising its favorable water-uptake attributes, leading to an approximately 50% enhancement in water-harvesting ability.

Zhang, Ma, and their teams took a different approach by introducing long-chain polyethylene glycol (PEG) into COF materials for the first time.^5^ They incorporated a PEG linear polymer containing 2,5-diethoxyterephthalohydrazid (DTH) molecules into the backbone of COF-42, which was based on the condensation of 1,3,5-triformylbenzene and DTH. Leveraging the long-chain structure and flexibility of the polymer, they successfully obtained defect-free, macroscale, and freestanding polyCOF membranes under ambient conditions. This study introduces a concept for fabricating a new class of advanced COF materials.

The substitution of ligands does not always “extend” the frameworks for the creation of large pores. Sometimes it can lead to defects in the original framework structure and alter the overall pore structure. Gu and Zhou’s team replaced the H_4_TBApy ligand (H_4_TBApy = 1,3,6,8-tetrakis(p-benzoic acid)pyrene) that has four coordination sites, with a bidentate ligand, [1,1′,3′,1″-terphenyl]-4,4″-dicarboxylic acid, in the NU-901 MOF.^6^ By controlling the ratio between the two ligands, they achieved precise regulation of defect sites in the MOF, allowing for a well-tuned ratio of mesopores to micropores. In a similar study, the research team led by Hailong Jiang placed UIO-66 MOF, with terephthalic acid as the ligand, in an acetic acid solution and subjected it to reflux heating.^7^ During the reflux process, acetic acid gradually replaced terephthalic acid, resulting in a significant number of defects and the formation of mesoporous structures, ultimately obtaining a hierarchical porous MOF.

## Engineering Novel Mesoporous Materials

Mesoporous materials are formed through the synergistic assembly of amphiphilic surfactants and precursors. Over the past two decades, a number of mesoporous materials with various compositions have been synthesized and reported.^8,9^ However, the construction of mesoporous metal oxides has remained a challenge. This is because the formation conditions for metal oxides are very demanding and often conflict with the synergistic assembly of surfactants in the soft-templating synthesis process. In the face of this challenge, the research team led by Prof. Yonghui Deng proposed a creative approach using heteropolyacids as precursors for synergistic assembly to construct mesoporous metal oxides.^10^ Due to the intrinsic micro-/nanostructures of heteropolyacids, their synergistic assembly with surfactants does not require the sol–gel process but involves an unusual self-assembly of organic–inorganic hybrid micelles. It results in the formation of novel topological mesoporous structures known as 3D orthogonally cross-stacked nanowire arrays. Based on this approach, they successfully prepared mesoporous Si-WO_3_ structures. Additionally, they successfully embedded Pt nanocrystals within the mesoporous frameworks, enhancing the activity of the mesoporous materials and laying the foundation for improved sensing performance.^11^

Similar to metal oxides, controlling the morphology of mesoporous noble metals presents significant challenges, which is due to the complexity and difficulty in tuning the crystalline nucleation kinetics of noble metal precursors while simultaneously maintaining the assembled mesoporous structures. To address this issue, the team led by Bin Liu utilized the “dual-template” characteristic of dioctadecyldimethylammonium chloride (DODAC), which can exist in both vesicular and rod-like micelle forms under specific conditions.^12^ They employed a one-pot synthesis approach to construct multimetal-doped hollow mesoporous Pd nanoparticles. By incorporating different metals and controlling reaction conditions, they were able to precisely tune both the hollow structure and pore architecture of these nanoparticles. This precise control synergistically enhanced the electrocatalytic performance for the electrochemical ethanol oxidation reaction.

## Engineering Pores Using Hard Templates

Pre-existing porous materials can also be employed as hard templates to construct entirely new porous materials. Although hard-templating offers advantages such as convenient synthesis and high universality, the resulting materials from the hard-template impregnation often exhibit bulk characteristics, and the ability to modify pore structures is limited compared to the soft-templating approach. However, in recent years, researchers have started to challenge these drawbacks based on a deep understanding of sol–gel chemistry.^13,14^

Kui Shen’s team impregnated a 3D ordered macroporous polystyrene replica template with a ZIF-8 precursor solution, resulting in the formation of macroporous ZIF-8.^15^ They discovered that the precursor solution concentration can precisely control the growth pattern and nanoarchitectures of hierarchical ZIF-8 single-crystals. The precursor concentration greatly influences the nucleation mechanism, leading to the formation of either spherical ZIF-8 aggregates or macroporous single-crystals. These different pore structures exhibit varying efficiencies in catalyzing the Knoevenagel reaction between benzaldehyde and malononitrile. Similarly, Ben Liu’s team impregnated a mesoporous silica framework with Pd precursor solution, leading to the synthesis of mesoporous Pd nanoparticles.^16^ As the authors gradually introduced boron (B) into the mesoporous Pd, they achieved a lattice transformation of metallic Pd from face-centered cubic to hexagonally close-packed without altering the pore structures.

In addition to using pre-existing hard-templates, the templates can also be generated *in situ*. Guihua Yu’s team reported the preparation of porous materials through a strategy called “freezing” of nanoparticle solutions.^17^ During the preparation process, the gradually forming ice crystals confines the assembly of nanoparticles in the 2D space between the ice crystals, resulting in the formation of numerous 2D layered structures. Upon removal of the ice crystals, these layered structures stack up to form a macroporous structure. This route exhibits high universality and can be utilized to prepare a range of porous structures with various compositions.

## Pore Structure Affects Mass Transfer

The construction of pore structures significantly affects the performance of porous materials, particularly the direct impact on substance adsorption and transport. Zhou’s team discovered that by controlling the assembly process, the same octahedral unit can be assembled into two different MOF structures: PCC-60 and PCC-67.^18^ In the former, the stacking of the assembly units forms a hierarchical pore structure with an interior-cage-pore size of 1.5 nm and an intercage stacking pore size of 2.4 nm. On the other hand, the latter is a single-pore MOF formed by dense packing of the cage units. They found that the hierarchical pores facilitate mass transfer within the superstructure, reducing the equilibrium time for adsorbing chiral substrates. As a result, PCC-60 with hierarchical pores can achieve remarkably higher enantiomeric excess values in separating racemates.

Feng Luo and colleagues carefully investigated the influence of pore environment on mass transfer.^19^ They modified a bipyridine-based MOF with coordination sites to introduce single-atom Cu^2+^ sites. These single-atom copper sites serve a dual function of catalyzing the conversion of nitrate ions into ammonia and storing ammonia, leading to superior performance in catalysis and gas storage applications. On the other hand, Jeffrey Long and his team used a newly developed molecule called BPP-7, which contains three phenyl rings and two carboxyl groups, to construct porous aromatic frameworks (PAFs).^20^ The resulting porous materials exhibit excellent stability due to the large number of benzene rings in the frameworks, resembling carbon materials. Additionally, the presence of numerous carboxyl groups enables effective separation of lanthanide/actinide elements.

In a recent work by Omar Yaghi’s team, they carefully investigated the influence of both pore structure and environment on water harvesting.^21^ They achieved this by transforming hydrazine-linked frameworks into hydrazide linkages within COFs and by altering the ligands to create 2D hexagonal (*hcb*), 2D square (*sql*), and 3D diamond lattice (*dia*) pore structures. Through systematic control and manipulation, they explored the impact of molecular-level changes on water adsorption, laying the foundation for the development of new water harvesting porous materials.

## Pore Structure Enhances Catalytic Performance

The presence of larger pore sizes and richer pore structures allows for better exposure of various active sites. When combined with improved mass transfer resulting from the porous structure, it enhances the overall performance of materials in catalysis and other applications.^22^ Dongyuan Zhao’s team successfully synthesized mesoporous TiO_2_ nanoparticles using an evaporation-induced self-assembly strategy.^23^ The resulting mesoporous FDU-19 exhibited an ultrahigh surface area (∼189 m^2^/g), large internal pore volume (0.56 cm^3^/g), and abundant defects (such as oxygen vacancies or unsaturated Ti^3+^ sites). They further demonstrated that by calcination in vacuum, the structure could be transformed into 2D ultrathin anatase single-crystal nanosheets dominated by nearly 90% exposed reactive (001) facets. Dye-sensitized solar cell tests showed that the mesoporous materials could achieve a photoconversion efficiency of 11.6%, surpassing the nonporous counterpart with the same crystal structure. In addition, Prof. Yonghui Deng and Dongyuan Zhao reported that Pt clusters can be homogeneously confined in the uniform spherical mesopores of mesoporous TiO_2_, which is based on the interaction between Pt nanoclusters and metal oxide pore walls. The loading facilitated the generation of interfacial active sites (Ti^3+^-O_v_-Pt^δ+^) during the reaction, thereby enhancing the cyclic catalytic activity.^24^

If the porous material itself lacks catalytic activity, large pores can still facilitate the loading of catalytic substances, especially for large biomolecules such as enzymes. Guangshan Zhu and colleagues employed a strategy of synergistic assembly using multiple ligands to construct framework materials with large pores.^25^ The synthesized frameworks exhibited pore sizes reaching 4–5 nm and an extremely high specific surface area of up to 2800 m^2^/g. With such large surface areas and pore sizes, they efficiently loaded lipase enzyme and maintained high catalytic activity of the loaded enzyme at different temperatures and pH levels.

The above content represents recent research on the construction and properties of pores, published in *ACS Central Science*. These works have made significant progress in addressing the challenges and have garnered widespread attention in the field, with many citing these articles in their own research. However, there are still many challenges in the structural control of porous materials: (1) Precise control of pore structures in framework materials: While there have been reports on constructing large or hierarchical pores by modifying ligands or using multiple ligands, universally applicable and guiding theories are still lacking.^26^ Determining whether new ligands can construct large or multilevel pore structures, or if they might disrupt the entire framework structure, often requires experimental trials. This significantly limits the synthesis and application of new framework porous materials. (2) Precise control of the coassembly process in mesoporous materials: One major challenge lies in achieving more accurate control over the coassembly process. Dongyuan Zhao’s team proposed using single micelles (monomicelles) from surfactants as assembly units to achieve more precise control over mesoporous assembly, yielding promising results.^27^ However, understanding the coassembly process requires not only improved synthesis control but also the application of more *in situ* characterization techniques. (3) Characterization of processes inside the pores: Another challenge lies in characterizing the processes occurring within the pores. Descriptions such as “higher surface area, more active sites, higher activity” or “more pores, faster mass transfer, better catalytic efficiency” are too general and do not facilitate deeper exploration and improvement of material performance. Additionally, some studies have shown counterintuitive phenomena occurring within the pores, highlighting the need for a deep understanding of the physicochemical reactions taking place inside the pores.

Overall, the world of pores is fascinating. The challenges of constructing pore structures, revealing the relationship between pore structures and properties, and designing high-performance, multifunctional, and reinforced noncovalent interaction materials based on pore structures are all exciting endeavors. We appreciate the opportunity to share this collection with you, and we hope it inspires your interest in porous materials as well. Let us delve into this research field full of infinite possibilities together.
